# Supplementary material for: Effects of Different Carbon Sources on Fumonisin Production and FUM Gene Expression by Fusarium proliferatum
Source: Toxins (Basel). 2019 May 22;11(5):289. doi: 10.3390/toxins11050289 (PMC6563204; doi:10.3390/toxins11050289)
Supplement: Supplementary file 1 [file toxins-11-00289-s001.pdf]

Supplementary Materials: Effects of Different Carbon Sources on Fumonisin Production and *FUM* Gene Expression by *Fusarium Proliferatum*

Yu Wu, Taotao Li, Liang Gong, Yong Wang and Yueming Jiang

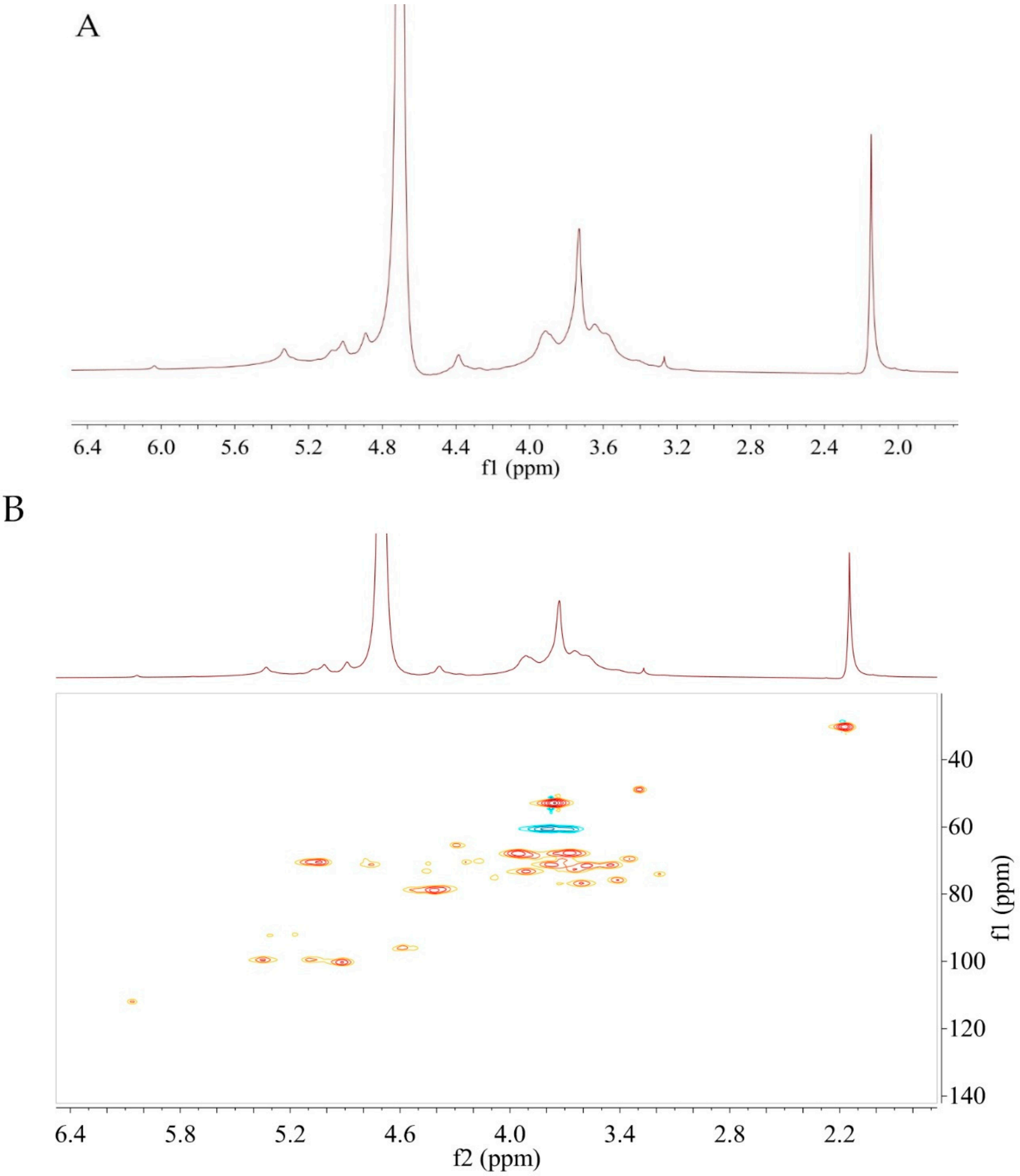

Figure S1. NMR spectra of unripe banana peel polysaccharides. (**A**) 1H spectra; (**B**) HSQC Spectra.

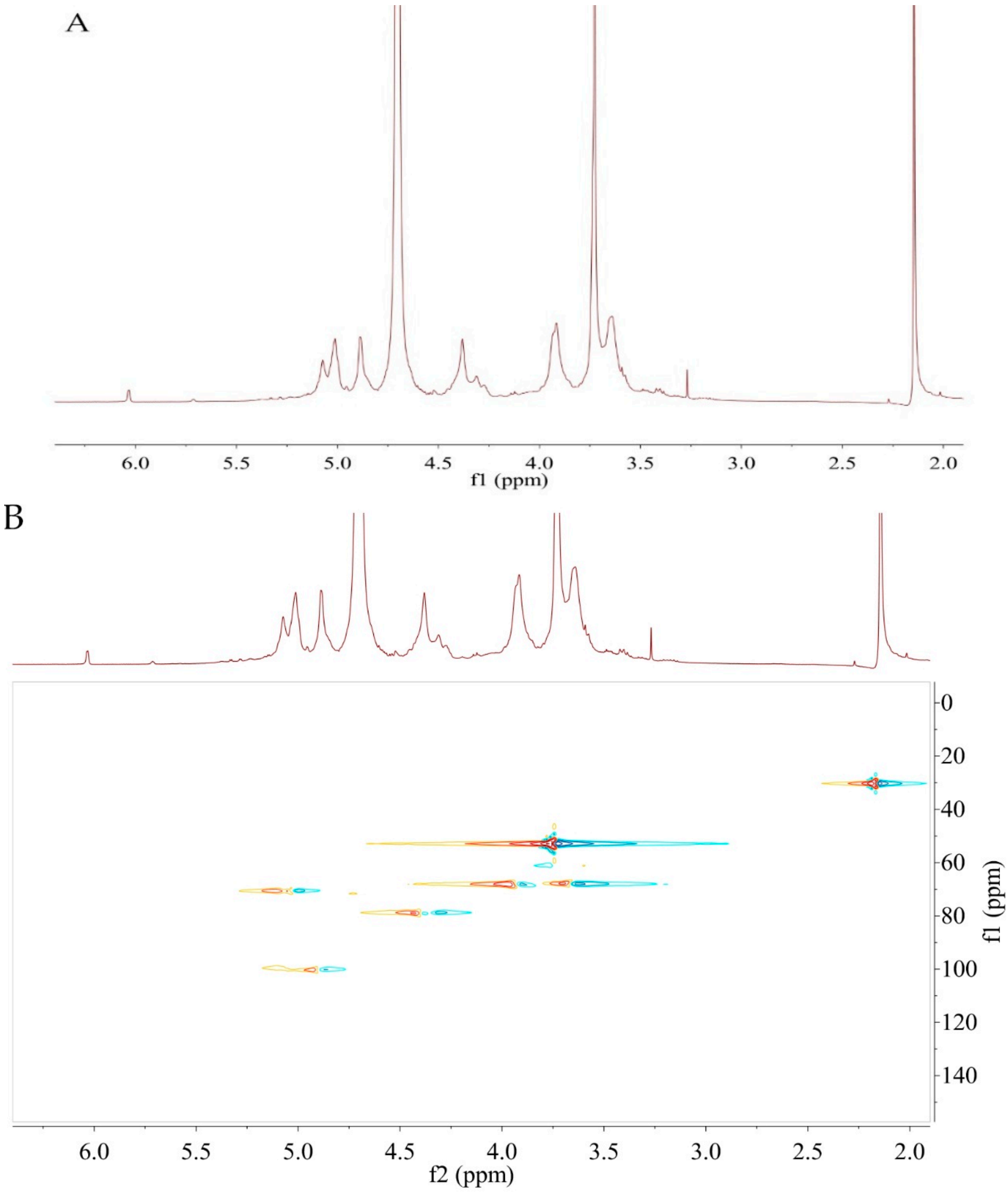

Figure S2. NMR spectra of ripe banana peel polysaccharides. (**A**) 1H spectra; (**B**) HSQC Spectra.
